# Clinical Features of and Risk Factors for Fatal Ebola Virus Disease, Moyamba District, Sierra Leone, December 2014–February 2015

**DOI:** 10.3201/eid2209.151621

**Published:** 2016-09

**Authors:** Yngvar Lunde Haaskjold, Håkon Angell Bolkan, Kurt Østhuus Krogh, James Jongopi, Karen Marie Lundeby, Sindre Mellesmo, Pedro San José Garcés, Ola Jøsendal, Åsmund Øpstad, Erling Svensen, Luis Matias Zabala Fuentes, Alfred Sandy Kamara, Melchor Riera, Javier Arranz, David P. Roberts, Paul D. Stamper, Paula Austin, Alfredo J. Moosa, Dennis Marke, Shoaib Hassan, Geir Egil Eide, Åse Berg, Bjørn Blomberg

**Affiliations:** Haukeland University Hospital, Bergen, Norway (Y.L. Haaskjold, O. Jøsendal, E. Svensen, G.E. Eide, B. Blomberg);; St. Olav Hospital, Trondheim, Norway (H.A. Bolkan, K.Ø. Krogh, S. Mellesmo);; Moyamba District Hospital, Moyamba, Sierra Leone (J. Jongopi, A.S. Kamara, A.J. Moosa, D. Marke);; Oslo University Hospital, Oslo, Norway (K.M. Lundeby);; Médicos del Mundo, Madrid, Spain (P. San José Garcés, L.M. Zabala Fuentes, J. Arranz);; Haraldsplass Diaconal Hospital, Bergen (Å. Øpstad);; University of Bergen, Bergen (E. Svensen, G.E. Eide, B. Blomberg);; Hospital Son Espases, Palma de Mallorca, Spain (M. Riera);; Instituto de Investigacion de Palma (IDISPA), Madrid (J. Arranz);; MRIGlobal, Rockville, Maryland, USA (D.P. Roberts, P.D. Stamper);; Sandia National Laboratories, Albuquerque, New Mexico, USA (P. Austin);; FELTP Public Health, Islamabad, Pakistan (S. Hassan);; Stavanger University Hospital, Stavanger, Norway (Å. Berg)

**Keywords:** Ebola hemorrhagic fever, Ebola virus disease, neglected diseases, Sierra Leone, viruses, fatal disease, risk factors, clinical features

## Abstract

The 2013–2016 outbreak of Ebola virus disease (EVD) in West Africa infected >28,000 people, including >11,000 who died, and disrupted social life in the region. We retrospectively studied clinical signs and symptoms and risk factors for fatal outcome among 31 Ebola virus–positive patients admitted to the Ebola Treatment Center in Moyamba District, Sierra Leone. We found a higher rate of bleeding manifestations than reported elsewhere during the outbreak. Significant predictors for death were shorter time from symptom onset to admission, male sex, high viral load on initial laboratory testing, severe pain, diarrhea, bloody feces, and development of other bleeding manifestations during hospitalization. These risk factors for death could be used to identify patients in need of more intensive medical support. The lack of fever in as many as one third of EVD cases may have implications for temperature-screening practices and case definitions.

## Abstract 2

Awareness of risk factors for death could help identify patients in need of more intensive medical support.

Since its discovery in 1976 (*1*), Ebola virus has caused small sporadic outbreaks with high case-fatality rates (CFRs), mostly in rural areas in central and eastern Africa (*2*). The outbreak of Ebola virus disease (EVD) in West Africa that began in Guinea in December 2013 (*3*,*4*) and subsequently spread to Sierra Leone and Liberia (*5*) is the largest known outbreak to date: >28,000 cases and >11,000 deaths were reported (*6*). Weak health systems, deep-rooted traditional burial customs, high population mobility, and early spread of disease in urban areas contributed to the unprecedented extent of the epidemic (*5*,*7*,*8*). The high death toll among healthcare workers further undermined weak existing health systems (*9*). Apart from Médecins Sans Frontiéres and a few other organizations, the international community responded slowly (*8*,*10*). However, a turning point came with a United Nations resolution on August 8, 2014, that led to creation of UNMEER (UN Mission for Ebola Emergency Response) on September 19. Leading public health agencies predicted that the epidemic could spiral out of control and estimated that, in a worst-case scenario, 1.4 million persons could become infected (*11*). In response, an increasing number of international stakeholders became involved, and several countries make direct monetary contributions. The epidemic peaked in September–October 2014 in Liberia and in December 2014 in Sierra Leone and Guinea; a gradual decline in the number of new cases followed (*6*,*8*).

The sporadic nature of previous Ebola outbreaks in remote areas of Africa has hampered the collection of clinical and laboratory data. In the 13 previously described outbreaks of Zaire EVD, the average CFR was 81% (1,123 deaths/1,390 cases) (*12*). The natural history of the 2013–2016 EVD outbreak in West Africa may vary from that in previous outbreaks. The World Health Organization (WHO) reported an overall CFR of 40% (11,314 deaths/28,634 cases) for the outbreak, which is probably an underestimate, and a CFR of 58% (513 deaths/881 cases) among healthcare workers (*6*). However, a large WHO-led study across the region found an average CFR of 71% (*13*). 

Specific treatment for EVD is not available, but aggressive supportive treatment has resulted in increased survival (*14*–*17*). Identification of patient groups with a higher risk for death could help target comprehensive supportive therapy to those most in need and could ultimately improve outcomes. Considering the evidence of Ebola virus persistence and delayed sexual transmission (*18*), late relapse of EVD in survivors (*19*), reemergence of EVD in Liberia 2 months after the country was declared Ebola-free (*20*), and the likely natural reservoir of Ebola virus in bats (*4*), it is imperative that health systems draw on lessons learned during the West Africa outbreak to prepare for future EVD outbreaks (*21*,*22*). We studied the clinical features of and risk factors for death among patients admitted to the Ebola treatment center (ETC) in Moyamba District, Sierra Leone, during mid-December 2014–March 2015.

## Methods

### Study Design

We performed a retrospective, descriptive study of clinical data from all patients admitted to the ETC in Moyamba District, Sierra Leone, one of the countries hardest hit by the West Africa Ebola epidemic. Moyamba District, located on the Atlantic Coast southeast of Freetown, the capital of Sierra Leone, has a rural population dispersed across 14 chiefdoms. The Moyamba ETC, one of 23 ETCs in the country, was established by the UK Department for International Development, administrated by the nongovernmental organization Médicos del Mundo, and manned by healthcare workers from Sierra Leone, Spain, France, United Kingdom, and Norway (*23*). We collected available data from all patients admitted to the ETC from its opening on December 19, 2014, until its closure on March 31, 2015; we obtained data on demographics, potential exposure situations, symptoms, findings, PCR test results, treatment, and outcome. The last patient with confirmed EVD was discharged on February 17, 2015. We retrospectively defined severe pain as pain clinically assessed to be severe enough as to lead the clinician to prescribe opiates. Laboratory resources (e.g., the ability to measure electrolytes) were not available at the ETC. Data were systematically compiled from multiple sources, including triage forms, patient records, and laboratory registries, and were plotted anonymously in EpiData 2.0 (EpiData Association, Odense, Denmark). The study was approved by the Sierra Leone Ethics and Scientific Review Committee (expedited review approved April 28, 2015) and the Western Norwegian Regional Committee for Medical and Health Research Ethics (reference no. 2015/538).

### Diagnostic Methods

Through January 11, 2015, diagnostic services (Ebola Zaire virus nucleoprotein real-time reverse transcription PCR [rRT-PCR]) were provided in Bo, Sierra Leone, by a US Centers for Disease Control and Prevention laboratory. On-site diagnostics were provided, beginning January 12, 2015, by the US Department of Defense (via the Defense Threat Reduction Agency’s Cooperative Biological Engagement Program) MEDaC (Moyamba Ebola Diagnostic Center) laboratory, which used 2 Ebola Zaire rRT-PCR assays provided by the Department of Defense Critical Reagent Program: a glycoprotein gene assay and a nucleoprotein gene assay (TaqMan minor groove protein binder). In addition, the laboratory ran a third PCR, the human RNase P assay, on every sample as a control for nucleic acid extraction and amplification. Genetic material was extracted robotically from whole blood by using the EZ1 Advanced XL instrument (QIAGEN, Hilden, Germany); rRT-PCR was subsequently performed using the AB 7500 Fast Dx Real-Time PCR Instrument (Applied Biosystems, Carlsbad, CA, USA). Positive template and negative extraction controls were included in every RT-PCR run; no-template controls for master mix and sample addition were also included. Negative controls during nucleic acid extraction and PCR set-up were used to screen for false positive stray template or potential contamination. This analysis provides semiquantitative results expressed as cycle thresholds (C_t_s). As requested from Ministry of Health and Sanitation, a malaria rapid test (SD. BIOLINE malaria Ag P.f [HRP-II]; Alere Standard Diagnostics, Yongin, South Korea) was added to the diagnostic workup for each patient beginning in February 2015.

### Statistical Analysis

We expressed the magnitude and statistical significance of risk factors as odds ratios with 95% CIs and p values; 2-tailed p<0.05 was used as the cutoff for statistical significance. Statistical analyses were performed in Stata 14 (StataCorp, College Station, TX, USA). To assess differences between proportions, we used the Fisher 2-tailed exact mid-p test (syntax file fishermidp.ado provided by M.W. Fagerland, Oslo Centre for Biostatistics and Epidemiology, Research Support Services, Oslo University Hospital, Oslo, Norway); to calculate odds ratios and 95% CIs, we used the Baptista–Pike or Cornfield mid-p interval with the mercii command. For comparison of continuous variables, including time variables, we used the 2-sample Fligner-Policello robust rank order test with the fprank command in Stata. For comparison of ordered groups (e.g., age groups), we used the nonparametric test for trend across ordered groups with the nptrend command in Stata. Kaplan–Meier survival plots were developed using the sts graph function in Stata.

## Results

### Patients

**Figure 1:**
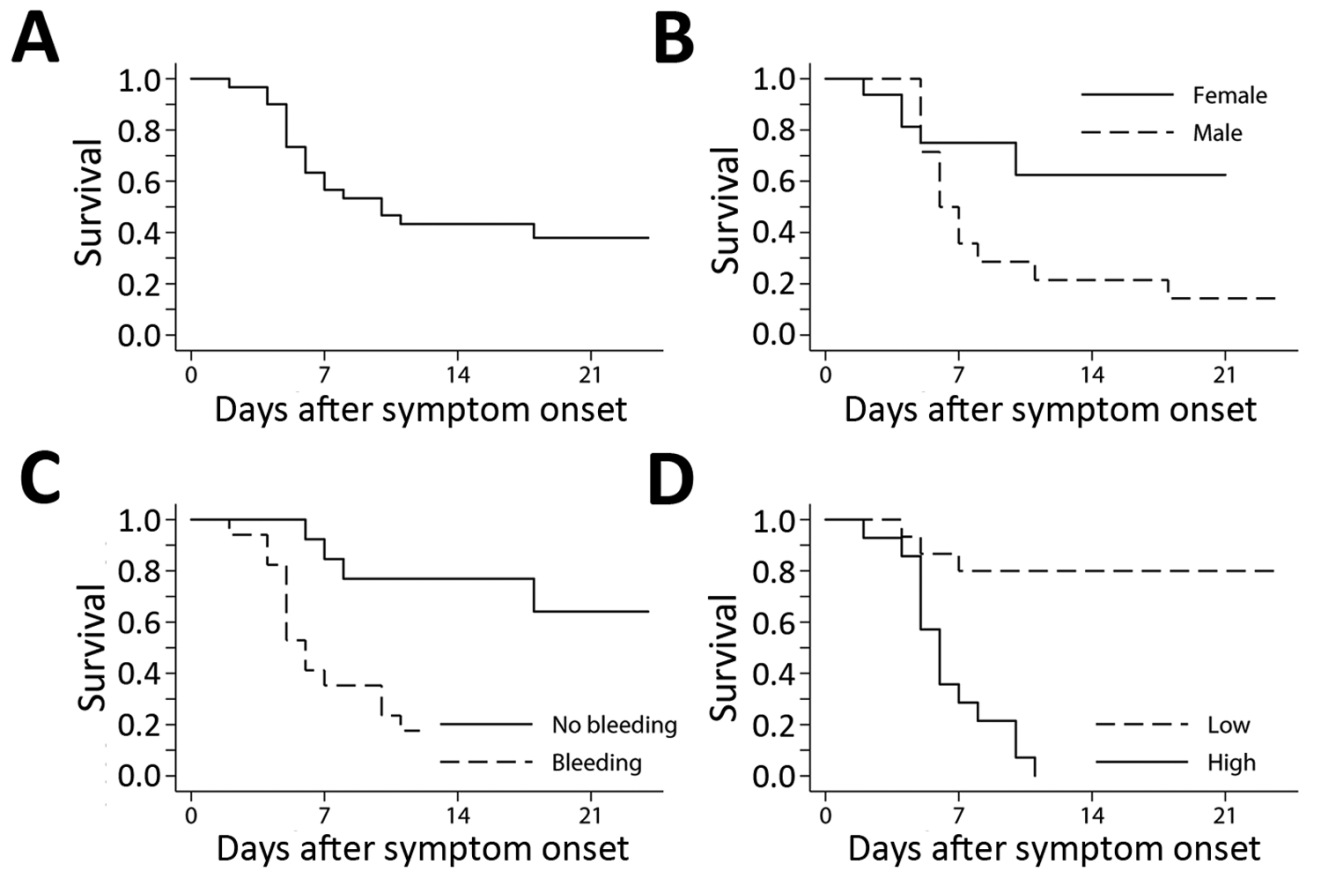
Survival analysis for patients with confirmed Ebola virus disease admitted to the treatment center in Moyamba District, Sierra Leone, December 19, 2014–February 17, 2015. Survival among A) all patients; B) male and female patients; C) patients with and without bleeding manifestations at admission; and D) patients with initial PCR results showing high- and low-level viremia, as defined by cycle thresholds <22 and >22, respectively.

We performed PCR on samples for 82 (93%) of the 88 patients admitted to the Moyamba ETC; 31 (38%) were positive for Ebola virus. Of these 31 patients, 28 (90%) reported contact with confirmed or suspected EVD case-patients, most of whom were household members (64%, 18/28); 11 (35%) had participated in burials for suspected EVD patients. No healthcare workers were among the 31 persons with confirmed EVD. Most of the Ebola virus–positive patients (87%, 27/31), including all who died, came from Ribbi chiefdom; 14 (45%) were male and 17 (55%) female (Table 1). Eighteen (58%) patients were 21–45 years of age (median 30 years, range 3 months–85 years). Overall CFR was 58% (18/31 died) (Figure 1, panel A), but the CFR was significantly higher among male than female patients (86% [12/14] vs. 35% [6/17]; p = 0.007) (Figure 1, panel B). No significant correlation was found between age and fatal outcome.

**Table 1:** Characteristics of patients with confirmed Ebola virus disease admitted to the treatment center in Moyamba District, Sierra Leone, December 19, 2014–February 17, 2015*

Characteristic	Patients†			OR (95% CI)	p value‡
	Total, N = 31	Died, n = 18	Survived, n = 13		
Demographic characteristics					
Sex					
M	14 (45)	12 (67)	2 (15)	11.0 (2.1–57.0)	0.007
F	17 (55)	6 (35)	11 (65)	0.009 (0.02–0.5)	
Age group, y					
<15	6 (19)	4 (22)	2 (15)	2.0 (0.25–16.3)	0.565§
15–44	19 (61)	11 (61)	8 (62)	1.4 (0.26–7.1)	NA
>45	6 (19)	3 (17)	3 (23)	1	NA
Healthcare workers	0 (0)	0	0	NA	NA
Length of incubation, median d (range)¶	8 (1–17)	7 (1–10)	8.5 (5–17)	NA	0.059#
Time from symptom onset to admission, median d (range)**	3 (0–23)	2 (0–17)	4.5 (1–23)	NA	0.006#
Time from symptom onset to death/discharge, median d (range)**	10 (2–45)	6 (2–18)	19.5 (12–45)	NA	<0.001#
Signs and symptoms					
Weakness	30 (97)	18 (100)	12 (92)	NA	0.210
Diarrhea	21 (68)	15 (83)	6 (46)	5.8 (1.2–25.0)	0.036
Fever	19 (61)	12 (67)	7 (54)	1.7 (0.42–7.4)	0.597
Loss of appetite	19 (61)	12 (67)	7 (54)	1.7 (0.42–7.4)	0.597
Vomiting	18 (58)	12 (67)	6 (46)	2.3 (0.60–11.0)	0.216
Red eyes	13 (42)	9 (50)	4 (31)	2.3 (0.55–8.3)	0.378
Nausea	8 (26)	4 (22)	4 (31)	0.64 (0.16–2.7)	0.551
Dysphagia	8 (26)	5 (28)	3 (23)	1.3 (0.22–5.7)	0.845
Hiccups	2 (6)	2 (11)	0	NA	0.332
Pain					
Overall	29 (94)	18 (100)	11 (85)	NA	0.084
Muscle pain	19 (61)	10 (56)	9 (69)	0.56 (0.15–2.2)	0.373
Joint pain	17 (55)	10 (56)	7 (54)	1.1 (0.27–4.1)	0.858
Headache	17 (55)	11 (61)	6 (46)	1.8 (0.48–7.7)	0.378
Abdominal pain	14 (45)	9 (50)	5 (38)	1.6 (0.42–7.3)	0.599
Chest pain	7 (23)	3 (17)	4 (31)	0.45 (0.10–2.1)	0.302
Pain requiring opiates	19 (61)	16 (89)	3 (23)	27 (3.9–144.0)	<0.001
Bleeding manifestations					
At admission	11 (35)	8 (44)	3 (23)	2.7 (0.61–11.0)	0.202
In feces	5 (16)	5 (28)	0	NA	0.033
From mouth	2 (6)	1 (6)	1 (8)	0.71 (0.04–14.0)	0.748
From eyes	1 (3)	0	1 (8)	0.0 (0–6.5)	0.210
From genitals	3 (10)	1 (6)	2 (15)	0.32 (0.02–3.2)	0.401
From puncture sites	1 (3)	1 (6)	0	NA	0.710
Any time during hospitalization	17 (55)	14 (78)	3 (23)	12 (2.3–50.0)	0.002
In feces	13 (42)	13 (72)	0	NA	<0.001
From mouth	9 (29)	8 (44)	1 (8)	9.6 (1.2–114.0)	0.031
From eyes	4 (13)	3 (17)	1 (8)	2.4 (0.31–33.0)	0.452
From genitals	6 (19)	4 (22)	2 (15)	1.6 (0.29–9.3)	0.838
From puncture sites	7 (23)	7 (39)	0	NA	0.019
Cycle threshold, median (range)††	22 (15.0–36.5)	20.5 (15–23)	26.5 (22.0–36.5)	NA	<0.001#

*NA, not applicable; OR, odds ratio. †Data are no. (%) except as indicated. ‡By Fisher 2-tailed exact mid-p test, except for ordered groups and continuous variables. §By nonparametric test for trend across ordered groups (i.e., age groups). ¶Incubation time was unknown for 8 patients with fatal and 3 with nonfatal disease. #By 2-sample Fligner-Policello robust rank order test for continuous variables (i.e., time variables, PCR values). **Time was unknown for 1 patient with nonfatal disease. ††Determined by real-time reverse transcription PCR; value was unknown for 1 patient with fatal disease.

### Incubation Period

Because of the retrospective study design and because patients in critical condition at admission could not give detailed histories, the time of exposure could be established for only 10 of the 18 patients who died and 10 of the 13 patients who survived. For those 20 patients, the median incubation period was 8 (range 1–17) days; no significant difference was found between fatal and nonfatal cases (Table 1). However, the median time from onset of symptoms to admission was significantly shorter for patients who died than for those who survived (p = 0.006). For the 18 patients who died, the median time from symptom onset to death was 6 (range 2–18) days; most (16/18 [89%]) died 4–11 days after symptom onset. EVD survivors were discharged a medium of 19.5 (range 12–45) days after symptom onset, when they were asymptomatic and had negative Ebola PCR results (C_t_ >36).

### Clinical Features

The most frequent symptoms among patients at admission were weakness (97%), diarrhea (68%), fever (62%), loss of appetite (62%), vomiting (58%), pain in muscles (62%) and joints (55%), headache (55%), abdominal pain (45%), and red eyes (42%) (Table 1). At admission, diarrhea was significantly more common among patients who died than those who survived (83% vs. 46%; p = 0.036).

Bleeding was present in 35% (11/31) of patients at admission and occurred in 55% (17/31) at any time during their hospital stay; bleeding occurred significantly more frequently among patients who died than those who survived (78% vs. 23%; p = 0.002) (Figure 1, panel C). Bloody feces was the most frequent hemorrhagic manifestation and a predictor of fatal outcome: 28% (5/18) of patients who died and none of those who survived had bloody feces at admission (p = 0.033), and 72% (13/18) of patients who died and none of those who survived had bloody feces at any time during their hospital stay (p<0.001). Bleeding from the mouth (p = 0.031) or puncture sites (p = 0.019) during hospitalization was also associated with death.

Pain was a prominent clinical feature and was often severe. All 18 patients who died reported pain, compared with 85% (11/13) of patients who survived (p = 0.084). The ETC was well stocked with analgesic medication, such as paracetamol and morphine, and did not experience shortages. Pain requiring opiate analgesia was significantly more frequent among patients who died than those who survived (89% [16/18] vs. 23% [3/13]; p<0.001). The mean daily morphine doses given to patients who eventually died (5.9 mg [range 2.3–15.0 mg]) and those who survived (4.4 mg [range 2.5–7.5 mg]) did not differ significantly (p = 0.175).

Major neurologic symptoms were infrequent. Among 4 patients with possible neurologic signs at or early after admission, 2 with hiccups died and 1 of 2 patients with seizures died.

### Laboratory Findings

**Figure 2:**
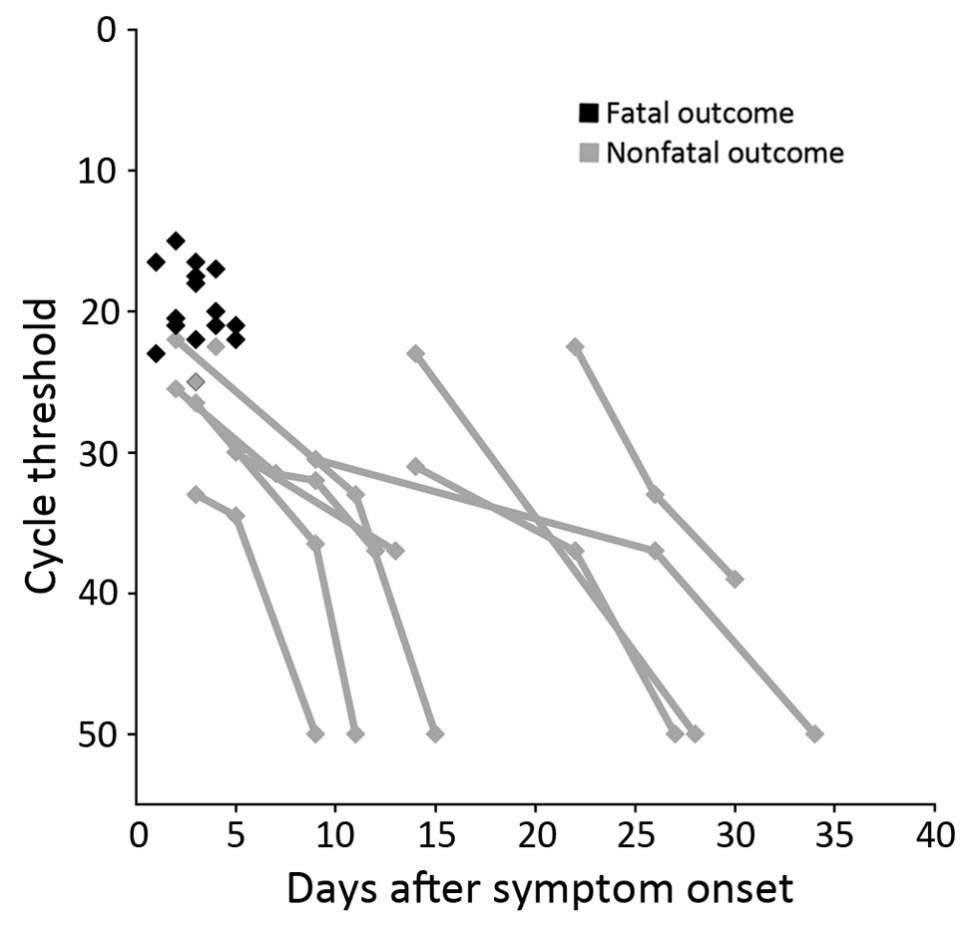
Ebola viral load for patients with confirmed Ebola virus disease admitted to the treatment center in Moyamba District, Sierra Leone, December 19, 2014–February 17, 2015. Viral loads were determined by semiquantitative PCR and are expressed as cycle thresholds for patients with fatal (n = 18) and nonfatal (n = 13) disease.

The median time from symptom onset to first Ebola PCR test did not differ significantly for patients who died (3.0 days) and those who survived (3.5 days) (p = 0.202). However, on the first PCR after admission, viremia was significantly higher in patients who died (median C_t_ 20.5 [range 15–23]) than those who survived (median C_t_ 26.5 [range 22–36.5]) (p<0.001) (Table 1; Figure 1, panel D; Figure 2). All patients with an initial C_t_ <22 (high viremia) died, but all patients with an initial C_t_ >23 (low viremia) survived.

### Treatment

Oral rehydration solution was administered liberally, and patients were encouraged to drink abundantly. Intravenous fluids were given to 26 (84%) of the 31 patients with confirmed EVD. In total, 29 (94%) patients received antimalarial treatment. Antimicrobial drugs were used empirically and administered to 26 (84%) patients with confirmed EVD. Among the patients who died, 78% (14/18) and 44% (8/18) received intravenous ceftriaxone and metronidazole, respectively. The difference in medications given to patients with fatal and nonfatal disease was not statistically significant (Table 2).

**Table 2:** Treatment given to patients with confirmed Ebola virus disease admitted to the treatment center in Moyamba District, Sierra Leone, December 19, 2014–February 17, 2015*

Treatment	No. (%) patients			OR (95% CI)	p value†
	Total, N = 31	Died, N = 18	Survived, N = 13		
Intravenous fluids	26 (84)	17 (94)	9 (69)	7.6 (0.90–97.0)	0.096
Antimalarial drugs	29 (94)	17 (94)	12 (92)	1.4 (0.07–28.0)	0.748
Ceftriaxone	23 (74)	14 (78)	9 (69)	1.6 (0.37–6.4)	0.551
Metronidazol	13. (42)	8 (44)	5 (38)	1.3 (0.33–5.8)	0.864
Albendazol	5 (16)	1 (6)	4 (31)	0.1 (0.01–1.1)	0.096
Zinc	29 (94)	17 (94)	12 (92)	1.4 (0.07–28.0)	0.748

*OR, odds ratio. †p values calculated by the Fisher 2-tailed exact mid-p test.

## Discussion

Our principal findings from this study were that EVD patients in Moyamba District had a higher rate of bleeding manifestations than reported elsewhere; a third of patients did not have fever at admission; and predictors for fatal outcome were shorter time from onset to admission, male sex, high viral load on initial laboratory test, severe pain, diarrhea or bloody feces at admission, and development of bleeding manifestations during hospital stay.

The main limitations of the study were the retrospective study design and the relatively small number of patients. Some data were incomplete because patients were in a critical condition when admitted and the hands-on time with patients was short because of infection control considerations. Cultural and linguistic differences among staff and patients may have represented a challenge for data collection and patient management. Laboratory testing was limited to PCR confirmation of EVD. In addition, the catchment area for the Moyamba ETC included some poorly accessible areas with strong traditional medicine influence (*24*), so ETC admissions could have been biased toward persons with severe disease and patients who sought care late in the disease course.

Strengths of the study were the availability of highly dedicated local staff; skilled international healthcare workers, including the almost continuous presence of medical doctors; availability of intravenous fluids and other supportive treatment; and consistent access to semiquantitative PCRs for Ebola virus testing. Due to the limited number of patients, clinicians could establish trusted associations with patients and meticulously register clinical features and responses to supportive treatment. The continuous presence of clinical staff enabled consistent data registration over time, decreasing the risk of registration bias.

Most patients were infected through contact with household members or burials for EVD patients. In contrast with findings from other treatment facilities during this and past outbreaks (*24*–*26*), none of the confirmed cases in Moyamba were among healthcare workers. The Moyamba ETC opened late in the epidemic; thus, lessons learned from other ETCs may have helped healthcare workers avoid virus transmission.

At the Moyamba ETC, the overall CFR was 58% for confirmed EVD patients; this rate is lower than that reported across Sierra Leone, Liberia, and Guinea (71% CFR) (*13*) but similar to that reported for healthcare workers in the region (*6*). The Moyamba ETC started operations in December 2014; at that late stage in the epidemic, most communities were better informed about EVD, and ill persons knew where to report for suspected EVD. Thus, unlike patients with mild symptoms earlier in the epidemic, patients with mild symptoms at this later stage may have sought healthcare. The Moyamba ETC was well equipped and staffed with competent local and international staff who could provide good care for the relatively few hospitalized patients. Of note, other ETCs, especially the one in Hastings, Sierra Leone, have documented CFRs as low as 23% in patients given comprehensive supportive treatment (*14*). However, the higher CFR in Moyamba could partly be explained by a bias favoring selection of severe cases from some of the less accessible areas that rely extensively on traditional medicine; in such areas, milder cases would have been treated locally rather than in Moyamba. Factors such as education level, cultural and socioeconomic factors, and health-seeking behavior may also have contributed to the differences in CFRs. 

No significant difference was seen in incubation time between fatal and nonfatal groups, but at admission, patients who eventually died had a clinical picture of rapidly progressing illness. The median time from symptom onset to admission was shorter in patients who died than in survivors. Most patients with fatal disease died 4–11 days after symptom onset. This time coincides with that observed in other West Africa centers during the outbreak (*17*,*26*,*27*). Young and old age have been associated with increased CFRs in other studies (*17*,*26*), but we found no such association. However, the mortality rate was higher among male (86%) than female (35%) patients (p = 0.007), consistent with the finding among male (40%) and female (29%) patients in Kerry Town, Sierra Leone (p = 0.181) (*27*). The reasons for this difference are unknown but might be due to an immunosuppressive effect of androgens and immunostimulatory effect of estrogen (*28*,*29*).

Fever is a major symptom in the WHO Ebola case definition, and thermometers have been used widely by healthcare workers and government officials to screen for EVD cases among persons in hospitals, shops, and airports and at road check points. However, as many as a third of the patients at the Moyamba ETC had no fever at admission. Several other reports have also noted the lack of fever among EVD patients (*30*,*31*), and there are indications that fever has been present in fewer EVD patients during the 2013–2016 West Africa outbreak compared with previous outbreaks (*1*,*24*). Most patients (61%) referred to the Moyamba ETC had negative Ebola PCR results, indicating that the WHO case definition is not sufficiently specific in identifying EVD cases and, consequently, Ebola-negative patients might be at risk for nosocomial Ebola virus transmission in ETC settings (*31*,*32*). A newly developed Ebola prediction score has shown promising results in risk-stratifying suspected EVD patients, but further validation is needed before this method can be put to use (*33*). The prediction score is based on 6 variables: contact with a sick person; presence of diarrhea, anorexia, or muscle pain; difficulty swallowing; and absence of abdominal pain. Clinical features alone are not sufficiently sensitive or specific to detect EVD cases, emphasizing the urgent need for an effective point-of-care test (*31*,*33*). A rapid diagnostic antigen test, which was recently tested in Sierra Leone, may become an efficient tool for excluding EVD among suspected cases in the future (*34*).

In general, bleeding manifestations were seen more frequently in the Moyamba patients than in those from other treatment facilities in Sierra Leone (*17*,*25*), but bleeding was less frequent in Moyamba patients than in patients during the 1976 outbreak (*1*). At Kenema Government Hospital in Kanema, Eastern Province, Sierra Leone, evidence of bleeding was found in only 1 of 106 EVD patients during the 2013–2016 outbreak (*17*). At the ETC in Moyamba, some of the patients with bleeding manifestations were from the same household and admitted during the same period. In the ETC, patients were not given any drugs that could have increased the risk of bleeding (e.g., nonsteroidal antiinflammatory drugs). However, we do not know if, before ETC admission, the patients took such medicines or any traditional medicine that might cause bleeding. Because of limitations on laboratory services, we had no information on whether patients might have concurrent diseases (e.g., malaria, Lassa fever, dengue fever) that might increase the tendency to bleed. In addition, many patients came from the same family, so we cannot exclude some form of genetic predisposition for bleeding manifestations. Furthermore, we lack knowledge about the different Ebola virus strains and routes of transmission in Moyamba District and cannot exclude the possibility that properties of the virus may have evolved during the epidemic.

Diarrhea and bloody feces on admission were significant risk factors for fatal outcome. Most patients who died (72%), but no survivors, experienced bloody feces during their hospital stay. The occurrence of bleeding manifestations overall as well as bleeding from the mouth or puncture sites during hospitalization also predicted fatal outcome. Bloody diarrhea may be attributable to severe enterocolitis caused by Ebola virus; disseminated intravascular coagulation; or concurrent bacterial, viral, or malarial infection (*24*,*35*). Chest pain was frequent and may suggest upper gastrointestinal tract involvement, particularly in combination with dysphagia. However, previous studies have suggested that pericarditis and myocarditis may cause chest pain in EVD (*24*). Although rhabdomyolysis has been postulated as a contributing factor to progressive renal failure and death in EVD (*17*,*27*), muscle pain was common in our study but was not a risk factor for death. Abdominal pain in EVD is probably of multifactorial etiology, and underlying pancreatitis has been proposed as a cause (*1*,*24*,*35*). Although a frequent finding, abdominal pain did not predict death in this study.

Almost all EVD patients reported pain, but severe pain requiring opiate treatment was significantly more frequent among patients with fatal disease. Attention should be given to palliation of severe pain in EVD. However, in this study, the short hands-on time with patients because of personal protection procedures limited the possibility of adequately treating pain with parenteral opiates. Transdermal administration of opiates, such as fentanyl-containing patches, was not available in the Moyamba ETC but would be a safer alternative for providing pain relief to EVD patients.

High viremia on admission was a strong predictor for death (100% fatality among patients with C_t_s <22), and low viremia was a good prognostic sign (100% survival among patients with C_t_s >23). This finding supports those from other studies (*17*,*24*,*26*,*27*,*36*,*37*) and should be kept in mind when interpreting trials of experimental treatments for EVD (*38*).

Systematic, comprehensive supportive therapy, including antimalarial, antimicrobial, and antihelmintic treatment, has been suggested to improve the prognosis for EVD patients (*14*,*27*). Although all patients admitted to the Moyamba ETC were treated according to the protocols developed by WHO and health authorities in Sierra Leone, the retrospective study design and the small sample size may have impeded the assessment of associations between treatment and outcome (*26*).

In summary, our findings are in agreement with those from other studies, but bleeding manifestations appeared to be more common in Moyamba than elsewhere and associated with fatal outcome. Awareness of risk factors for death, including short time from symptom onset to admission, male sex, diarrhea, bloody feces and other bleeding manifestations, severe pain, and high viral load, could be used to group patients at greatest risk into dedicated wards with more intensive medical support. Selective use of intravenous fluid therapy could be a rational approach when resource constraints and infection control considerations prevent delivery of fluid therapy to all patients. Severe pain was common, particularly among moribund patients, calling attention to the need for adequate and safe pain relief (e.g., with transdermal administration of opiates) for EVD patients. The lack of fever in as much as one third of EVD patients and the finding that 61% of admitted patients tested negative for EVD may have implications for screening practices, case definitions, and isolation strategies. The sharing of clinical experiences regarding EVD, a hitherto rare disease, may help prepare for more effective patient care in future outbreaks.
